# A review of advances in the treatment of diabetic nephropathy by modulating intestinal flora with natural products of traditional Chinese medicine

**DOI:** 10.3389/fphar.2025.1628294

**Published:** 2025-10-01

**Authors:** Tao Wang, Ming Chen, Jiwen Xu, Yuhang Qiao, Xinhui Huang, Xiaoming Yan, Fuli Zhang

**Affiliations:** ^1^ Graduate School, Heilongjiang University of Chinese Medicine, Harbin, Heilongjiang, China; ^2^ Department of Nephrology, Heilongjiang Academy of Chinese Medicine Sciences, Harbin, Heilongjiang, China; ^3^ School of Basic Medical Sciences, Heilongjiang University of Chinese Medicine, Harbin, Heilongjiang, China; ^4^ Hepatobiliary Spleen and Stomach Disease Department, Heilongjiang Academy of Chinese Medicine Sciences, Harbin, Heilongjiang, China

**Keywords:** diabetic nephropathy, intestinal flora, natural products, TCM, metabolite

## Abstract

Diabetic Nephropathy (DN) is one of the most serious complications of diabetes mellitus (DM). Hemodynamic irregularities, metabolic problems, and hormone production are only a few of the many mediators and routes that contribute to the intricate and varied genesis of DN. DN is the most common cause of end-stage renal disease, which is characterised by persistently low glomerular filtration capacity and hyperglycaemia. Numerous studies have been conducted recently on the function of gut flora in DN. According to current research, the formation and progression of DN have been linked to imbalances in the gut microbiota and abnormal microbial metabolite production. Therefore, intestinal flora modulation may be an entry point for the treatment of DN. As an increasing number of studies are using natural products of traditional Chinese medicine (TCM) as a therapeutic tool, this article reviews the progress of TCM natural products in treating DN by modulating gut microbiota.

## 1 Introduction

Diabetic nephropathy (DN), as a common cause of end-stage renal disease, is a microvascular complication of diabetes mellitus (DM) and is one of the fastest-growing public health issues in both developed and developing countries. Globally, the recent notable rise in DN cases is having an impact, frequently resulting in increased morbidity and mortality in DM patients and driving up healthcare costs and other related expenses. And smoking, environmental factors, and hyperlipidemia are prominent causes of DN (Papadopoulou-Marketou et al., 2018). Currently, DN can be classified into four categories (Ⅰ, Ⅱ, III, Ⅳ) via glomerular lesions at the time of biopsy (Tervaert et al., 2010). As a typical early sign of DN, microalbuminuria is frequently detected in clinical practice to facilitate early screening. Reducing proteinuria is also a key component of the diagnosis and treatment of DN (Samsu, 2021). As for the treatment method, renin-angiotensin system (RAS) inhibitors, angiotensin receptor blockers, and aldosterone receptor antagonists are the main treatments for DN. Research has shown that the complement system is also crucial for both diagnosis and treatment of DN (Liu et al., 2022). However, even with proactive treatment measures, it is still difficult to prevent the progression of DN completely. The occidental treatments are always unable to achieve the desired effect and have obvious toxic side effects. As a result, ideal and safe treatment are still needed to control the course of DN.

With the accumulating research of DN, it was found that gut microbiota plays a critical role in DM and DN, and the precise effects and mechanisms are continuously excavated (Flyvbjerg, 2017). The gut microbiota is a diverse collection of bacteria, fungi, archaea, and other microorganisms that coexist with their host. Several studies have demonstrated that the gut microbiota will alter as the host ages, which has significant effects on the host’s metabolism, neurological system, immune system, and mental health, among other systems (Adak and Khan, 2019). In addition to changes in composition, the function of gut microbiota varies and is regulated by different factors. Studies show that lipids in food directly affect the growth and metabolism of bacteria. Furthermore, the gut flora can affect the host’s level of lipid metabolism and result in a number of diseases with aberrant blood lipid metabolism (Schoeler and Caesar, 2019). According to studies on a variety of animals, the gut microbiota may help the host in its early years, but as the host matures, the negative effects of the microbiota also become more noticeable, and the microbiota may even accelerate the aging process (Maynard and Weinkove, 2018).

With the development of contemporary science and technology, the role of microbiota in various diseases is proved by numerous research and continues to intensify, such as gut microbiota, urine microbiota. Currently, gut microbiota is one of the most extensively studied microbiota and has been discovered to affect the digestive system, urinary system and even some mental health conditions like autism and depression (Mangiola et al., 2016). Furthermore, the gut-kidney axis has been shown in several studies to be influenced by the gut and microorganisms, and interventions in the gut barrier and microorganisms may slow the progression of kidney disease. Dysregulation of the gut microbiota has been observed in DN patients, which are always accompanied by increased uremic solutes and decreased short-chain fatty acids (Ma et al., 2022). Accordingly, gut microbiota is an important target of DN, and ideal regulation methods on gut microbiota are needed.

Traditional Chinese medicine (TCM) has been used for hundreds of years to treat DM and DN, has garnered significant benefits in recent years (Liu et al., 2022). Under the theory of TCM, DM is known as “thirst-quenching” with the symptoms of polydipsia, polyuria, and polyphagia. The basic yin deficit and fire etiology of the condition cause these symptoms. TCM treatment has the advantages of multi-target, overall regulation, and no significant toxic side effects. At present, clinical treatment on DN is carried out from generating fluid and nourishing blood, detoxifying and detumescent, and nourishing Qi and Yin. More research present that the use of TCM interventions ameliorate DN by addressing the gut microbiota. A thorough examination of the therapeutic approach is carried out and shows promising outcomes. Therefore, this review summarized the effects of gut microbiota in DN development, and the progress of TCM and natural ingredients of TCM in treating DN by modulating gut microbiota.

## 2 Materials and methods

Questions were designed using the main research question of this study as an outline: changes in intestinal flora in animal models of DN lesions and human patients compared to normal controls. To study the role of the gut-renal axis in kidney disease (with DN as the main disease); investigate the modulation of the enterorenal axis by gut microbial metabolites that change significantly in the DN state compared to normal controls; organize and summarize herbal compounds, unit herbs, and monomers that moderate DN status by modulating changes in the gut flora and its metabolites. English databases (Pubmed, web of sciences) and Chinese databases (CNKI, Wanfang Database, Vip). The search strategy is: (Topic = ‘gut flora’ OR ‘Intestinal flora’) AND (Topic = ‘Metabolites’) AND (Topic = ‘diabetic kidney disease’ OR ‘diabetic nephropathy’) AND (Topic = ‘Natural Products’ OR ‘Traditional Chinese Medicine’ OR ‘Chinese Medicine Compounding’). And searching years are from 2014 to 2024).

## 3 The role of the gut microbiota on DN

DN is a common consequence of DM affecting the small blood vessels (Lee et al., 2021). Additionally, it is essential for preserving digestive homeostasis and controlling metabolism. The dynamic and diverse microbiota present in the human gut is intimately related to both host health and development of DN (Thursby and Juge, 2017).

### 3.1 Gut microbes affecting kidney disease

Changes in Firmicutes, Proteobacteria, and Bacteroidetes are dominant in DN vs. non-DN (He et al., 2022). A prominent marker of DKD is the low ratio of Firmicutes (Gram-positive)/Bacteroidetes (Gram-negative) (Zaky et al., 2021; Grasset et al., 2017). The stool of patients with diabetic nephropathy shows a relative increase in *Bacteroides* stercoris, Verrucomicrobia, Proteobacteria, Fusobacteria (Lin et al., 2022), *Haemophilus*, Escherichia–Shigella, Megalococcus, Veillonella, and Anaerostipes (Du X. et al., 2021), and a relative decrease in butyrate-producing bacteria (Roseburia, Faecalibacterium, *Clostridium*, Coprococcus, and Prevotella) (Jiang et al., 2017) as well as in potential probiotic genera (Trichella spp., Enterobacteriaceae, *Lactobacillus*, and Bifidobacterium). Additionally, a significant association between creatinine and *Clostridium* was seen in the stool of patients with DN, highlighting the significance of gut microbiota for these individuals (Zhang L. et al., 2022). In addition to validating the relationship between gut microbiota and DN in biological models such as mice, Mendelian randomization analyses have revealed a significant association between Errucomicrobiae, Verrucomicrobiales, and Verrucomicrobiaceae and DN (Yan et al., 2024). Later studies showed that the genera Terrisporobacter and Lachnospiraceae UCG008 were associated with the severity of DN, whereas the phylum Proteobacteria and Dialister may protect the condition (Fang et al., 2024). These studies help to understand the relationship between DN and gut microbiota.

### 3.2 Gut-kidney axis

**FIGURE 1:**
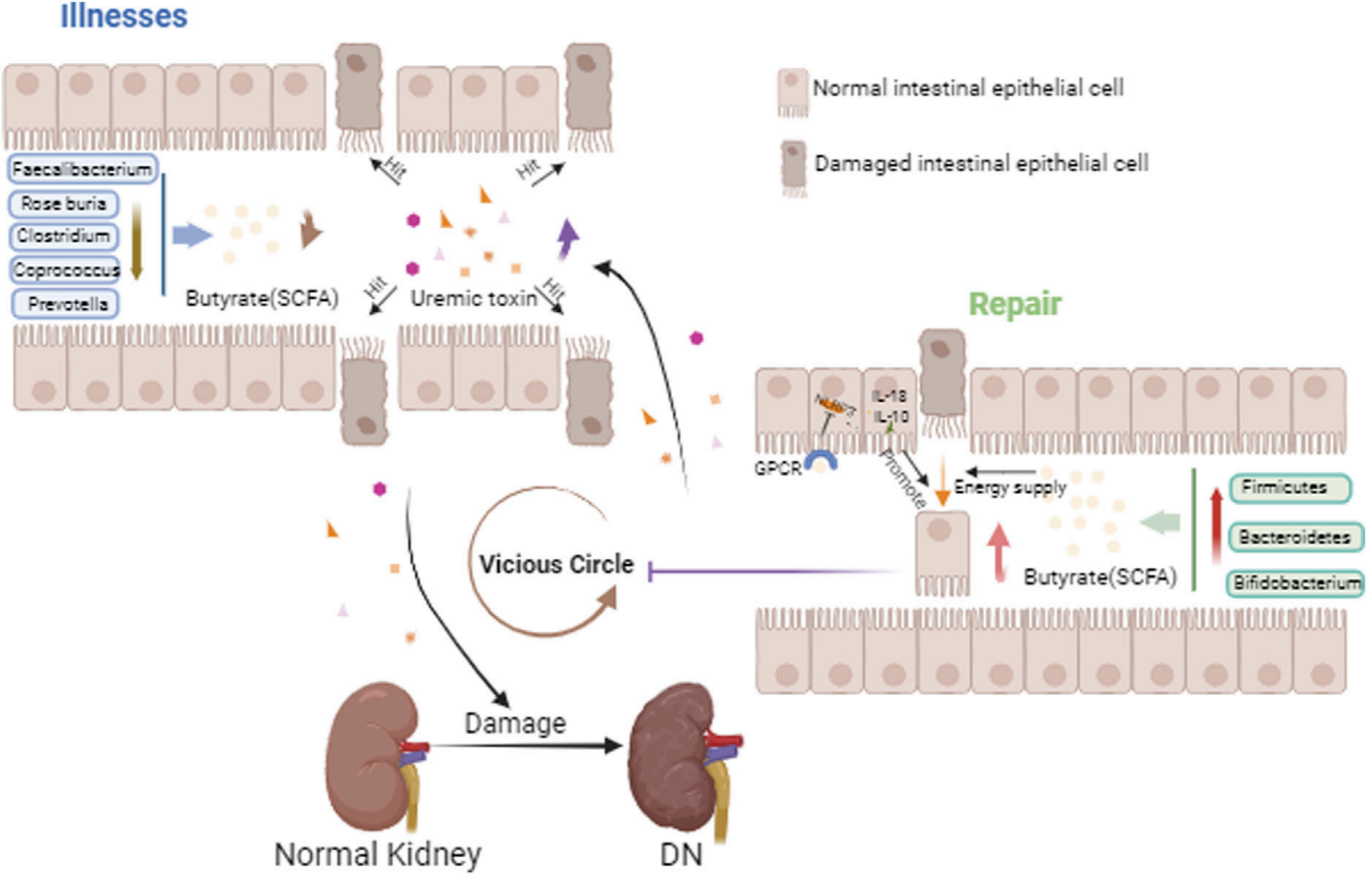
Gut-kidney axis in DN. This figure mainly depicts the reduced distribution of butyric acid-producing bacteria in the gut in the DN state, the reduction of butyric acid, the damage to the intestinal epithelium, the entry of accumulated uremic toxins into the circulation aggravating renal injury, and with the aggravation of renal injury, the uremic toxins are deposited in the gut, becoming a vicious cycle. And with the increase of butyric acid producing bacteria, this vicious cycle is inhibited.

The intestinal epithelial cells have a crucial role in the intestinal tract. On the one hand, they have pattern recognition receptors (PRRs) on their surface that recognize the microbial cell wall and thus activate the immune response and the inflammatory response. For example, toll-like receptor (TLR) is a type of PRRs, which activates phagocytes Th17, Th1 and thus stimulates the release of pro-inflammatory cytokines by recognizing segmented filamentous bacteria and *Salmonella* (Seo et al., 2015). On the other hand, the intestinal epithelial cells are also a barrier. When various pathogenic factors cause damage to the intestinal epithelial cells, the intestinal barrier is damaged and the permeability of the intestinal barrier is increased, which in turn invades the circulation and exacerbates intestinal inflammation, in a process known as “gut leaky” (Paray et al., 2020). In healthy individuals, dietary proteins are metabolized to produce uremic solutes, including p-Cresyl sulfate (PCS), indoxyl sulfonate (IS), indole-3-acetic acid, trimethylamine N-oxide (TMAO), and phenylacetylglutamine (PAGIn) (Amini Khiabani et al., 2023; Chen et al., 2019). Butyrate decreases serum levels of renal inflammatory and uremic toxins, whereas end-stage renal disease (ESRD) patients with end-stage testing of flora found a reduction in butyrate-producing bacteria, Roseburia, Faecalibacterium, *Clostridium*, Coprococcus, and Prevotella (Jiang et al., 2017; Li et al., 2022). Meanwhile, within this disease state, uremic toxins accumulate in large quantities, with the decline of renal excretory function, and the colon becomes the main part of the elimination of urea and uric acid. This process will lead to an increase in the pH value of intestinal fluids, which disrupts the intestinal homeostasis. Consequently, the microbiota changes, further aggravating the accumulation of uremic toxins (Hatch and Vaziri, 1994; Bourke et al., 1966). It has been shown that continued exposure of colonic epithelial cells to urea disrupts their barrier protection, resulting patients in “gut leaky” with increased levels of systemic endotoxins and bacterial products leading to chronic, systemic inflammation (Vaziri et al., 2012; Vaziri et al., 2013). In conclusion, the course of kidney disease affects the damage to the intestinal barrier, which exacerbates the progression of kidney disease, creating a vicious circle (Figure 1). DN is the main cause of ESRD in most developed countries (Umanath and Lewis, 2018). Therefore, in order to slow down the process of evolving into ESRD in the DN state, intervention of the gut microbiota and its metabolites is necessary.

## 4 Effect of gut microbiota metabolites on DN

**FIGURE 2:**
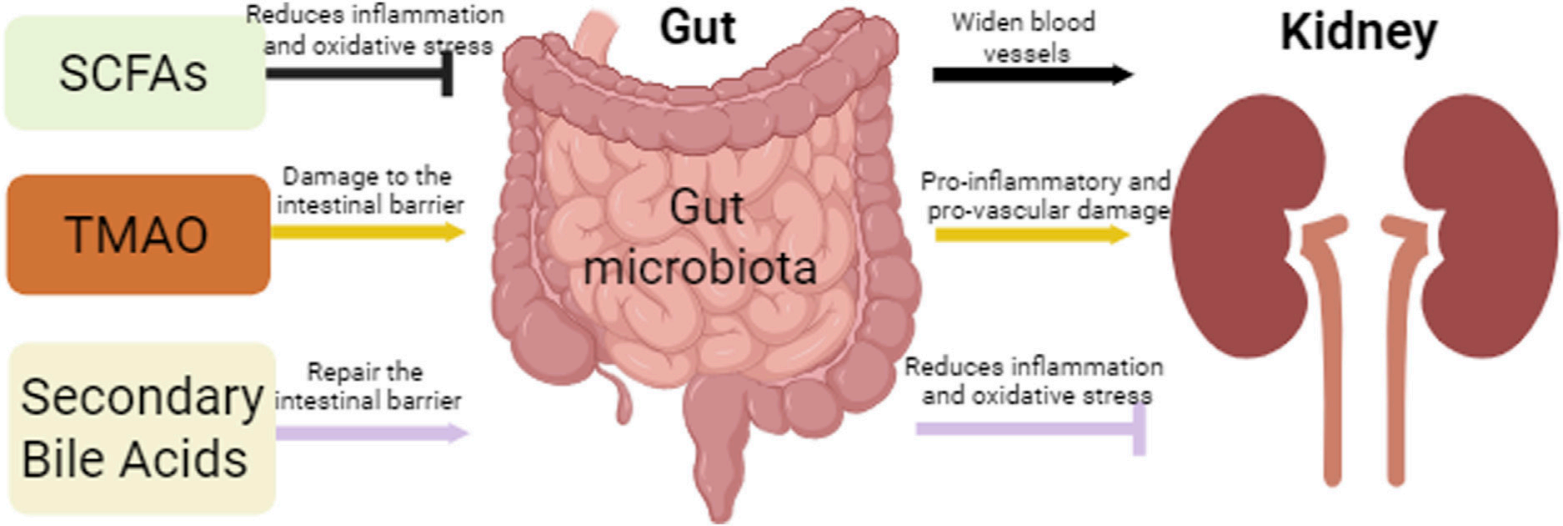
Intestinal and renal effects by gut microbiota metabolites. This figure depicts the effects of three major microbial metabolites, SCFAs, TMAO, and secondary bile acids, on the gut and kidneys, where SCFAs and Secondary Bile Acids protect the gut and kidneys by attenuating oxidative stress and inflammation, while TMAO promotes the inflammatory process leading to renal fibrosis, while the TMAO accumulation leads to damage of the intestinal epithelial barrier.

Short-chain fatty acids (SCFA), trimethylamine N-oxide (TMAO), and secondary bile acids are three gut microbial metabolites that are essential for the regulation of the gut microbiota (Tian et al., 2023) (Figure 2).

### 4.1 SCFAs

Dietary fiber and resistant starch are fermented by gut microbes to form metabolites known as SCFAs, which are primarily acetic, propionic, and butyric acids. SCFAs play an important role in regulating host metabolic health and immune response. SCFAs can reduce intestinal and renal inflammatory responses, which is particularly important in patients with DN. As chronic inflammation, SCFAs is a key factor in the progression of DN, with resisting oxidative stress by propionate and butyrate, and exacerbating the progression of DN by acetate (Tian et al., 2023). SCFAs can be used as an energy source for intestinal cells, aiming to improve metabolic status, and participate in the regulation of blood lipid levels and blood pressure regulation, which can help lower blood pressure and reduce the risk of cardiovascular disease. In contrast, SCFAs can improve insulin sensitivity and help lower blood sugar levels, in the interest of patients with DN. Furthermore, SCFAs with vasodilating properties can enhance renal function by increasing blood flow to the kidneys (Zaky et al., 2021; Deng et al., 2022; Li et al., 2020; Tang et al., 2022). SCFAs are also extensively involved in the immune response, such as stimulating the production of IL-18 by binding to G protein-coupled receptors, and thus promoting the repair of the intestinal epithelial barrier (Macia et al., 2015). SCFAs also enhance phagocytosis by neutrophils and inhibit the production of inflammatory mediators by macrophages (Vinolo et al., 2009; Chang et al., 2014). In conclusion, gut microbes can maintain the integrity of the intestinal barrier by modulating the immune response through the production of SCFAs, which further may slow down the progression of kidney disease. Inulin-type fructans (ITFs) can shield kidney injury in db/db mice by producing gut bacteria that control SCFAs (Luo et al., 2022). On the other hand, empagliflozin can mitigate kidney damage in DN-affected mice by elevating the SCFA-producing bacteria Odoribacter and Bateroid while lowering the LPS-producing Oscillibacter (Deng et al., 2022).

### 4.2 TMAO

Trimethylamine (TMA) is mostly oxidized by gut microbes, and can produce TMAO. Ingested nutrients like choline and lecithin are broken down by gut microorganisms to create TMA, then TMA transported to the liver via the portal vein circulation and converted to TMAO. Patients with diabetes may produce more TMA due to an imbalance in their gut microbiota, which raises the blood level of TMAO. Furthermore, it has been discovered that TMAO increases platelet hyperresponsiveness, inhibits cholesterol reverse transport, and encourages the development of macrophage foam cells, all of which may aid in the development of endothelial dysfunction and atherosclerosis to causes diabetic microvascular damage. TMAO can accelerate the development of DN by activating inflammatory pathways, such as NLRP3 inflammasome signaling pathway and nuclear factor kappa B (NF-κB) signaling pathway, leading to the release of inflammatory factors and promoting tubulointerstitial injury and renal fibrosis. Therefore, chronic inflammation and TMAO may play a significant role in the development of DN (Tian et al., 2023; Tanase et al., 2020; Kalim and Rhee, 2017; Huo et al., 2023; Fang et al., 2021). On the whole, the gut microbial metabolites mentioned above have a significant impact on renal function and metabolism in addition to being engaged in inflammatory and immunological responses in diabetic nephropathy. Regulating the gut microbiota may help improve the condition of people with DN.

### 4.3 Secondary bile acids

The metabolism of primary bile acids by gut microbes results in the production of secondary bile acids, which are involved in controlling the host’s immunological and metabolic reactions. In addition to inducing renal fibrosis and inflammation through the NF-κB and other inflammatory signaling pathways being activated, gut microorganisms can also create secondary bile acids that support renal integrity of the intestinal barrier, stop the growth of bacteria in the gut and their byproducts (e.g., urotoxins) from entering the bloodstream, and reduce the burden on the kidneys (Lu et al., 2018). Secondary bile acids are also good at regulating metabolism, and they may have a positive impact on metabolic control in DN patients by binding to receptors in host cells, such as farnesoid X receptor (FXR), which are involved in regulating host metabolic processes, including lipid, glucose, and energy metabolism (Yang et al., 2021). As an illustration, it raises muscle energy expenditure and, through encouraging GLP-1 (glucagon-like peptide-1) release, may enhance insulin resistance and blood glucose metabolism. Ursodeoxycholic acid (UDCA) is a secondary bile acid. And it was reported that UDCA has shown a protective effect on the kidneys of diabetic rats by decreasing SGLT2 expression and reducing oxidative stress, which provides a new potential avenue for the treatment of DN (Osorio et al., 2012).

## 5 TCM natural products regulate gut microbiota in the treatment of diabetic nephropathy

High blood glucose and issues with glucose metabolism are common in models and people with DN. These issues thicken the glomerular basement membrane and result in tubulointerstitial fibrosis (Forst et al., 2022). These conditions worsen renal damage and reduced rate of glomerular filtration (Forst et al., 2022) managing the underlying causes of hypertension and diabetes, such as aldosterone receptor antagonists, renin-angiotensin system inhibitors, and angiotensin receptor blockers, is currently the main treatment for DN in western medicine (Liu et al., 2022). The intimate relationship between gut microbiota and DN has been demonstrated, as well as the possible contribution of certain bacteria to the emergence of glucose dystrophy or insulin resistance. With the development of the gut-kidney axis theory, an increasing number of medical experts are becoming aware of the advantages of TCM for DN treatment. Specifically, TCM has a therapeutic effect on DN through regulating the gut microbiota and its metabolites.

### 5.1 Traditional Chinese medicine prescriptions

TCM prescription is one of the important forms of TCM treatment, which is also essential in DN therapy (Table 1; Table 2). Xiancao Granule (CXCG) is a traditional Zhuang medicine prescription derived from Zhuang folk herbs in Guangxi, China. The Zhuang are an ethnic minority in China with a rich ethnic medicine culture and traditions. Mo et al. (2024) showed that CXCG could increase the relative abundance of beneficial bacteria (e.g., Alloprevotella, Oscillibacter, Anaeroplasma, and Anaerotruncus) and decrease the relative abundance of harmful bacteria (e.g., Faecalibacterium), improving the structure of intestinal microbiota in DN rats. By increasing the abundance of gut microbiota with anti-inflammatory properties, CXCG may help reduce inflammatory markers associated with DN, such as serum levels of IL-6, TNF-α, and MCP-1. In addition, it can also improve the metabolic disorders of DN rats by regulating metabolic pathways related to carbohydrate and amino acid metabolism, such as TCA cycle, vitamin C and aldehyde metabolism, and C-type lectin receptor signaling pathway. The microbiota and metabolism were regulated to improve the renal function parameters of DN rats, including decreased levels of urine microalbumin (MUA) and serum creatinine (Scr), as well as less degenerative kidney tissue damage. Qing-Re-Xiao-Zheng formula (QRXZF) is a TCM prescription (Wang et al., 2023; Gao et al., 2021) that reduced the release of inflammatory factors by inhibiting the activation of inflammatory signaling pathways such as TLR4/NF-κB/NLRP3. Meanwhile, QRXZF reduced renal inflammatory response by enhancing intestinal barrier function, reducing intestinal permeability, translocation of endotoxins (such as lipopolysaccharide LPS), and the production of inflammatory factors. Among them, polysaccharides and other ingredients not only have antioxidant effects, but also scavenge free radicals, reduce oxidative stress, and protect kidney cells. Moreover, QRXZF enhanced the balance of intestinal microecology and accomplished the goal of treating diseases by encouraging the growth of helpful bacteria and preventing the reproduction of bad bacteria through the metabolism of intestinal microorganisms. According to Hong et al. (2023) it lessened the buildup of urinary toxins such as trimethylamine-N-oxide, p-cresyl sulfate, and indoxyl sulfate. It also lessened inflammation by lowering levels of inflammatory cytokines like NLRP3, IL-6, and IL-17A. In addition, its active ingredient, calycosin-7-O-β-D-glucoside, interacted with the gut microbiota and stimulated the growth of probiotics, thus exerting its therapeutic effect. Besides, it was reported that Jingui Kidney Qi Pills, Ginseng Qi Dihuang Decoction, Shrinking Spring Yi Kidney Fang, Yuye Tang, Bekhogainsam Decoction, and Jowiseungki decoction (JSD) have similar regulatory mechanisms (Zhang et al., 2024; Meng et al., 2020a; Meng et al., 2020b; Guo et al., 2024; Du X. M. et al., 2021; Yao et al., 2020). And Yuye Tang also improved intestinal barrier function, lower intestinal permeability, and raised the expression levels of the colonic mucosal barrier proteins Occludin and ZO-1, which stopped bacteria and toxins from translocating. Reducing the risk of systemic inflammation and organ damage by regulating the levels of cAMP and cGMP, Yuye Tang affected the body’s energy balance and metabolic processes, which might help to improve energy metabolism and overall health in patients with DN. Xiaoli Wang, et al. noted that nephritis rehabilitation granules (Wang et al., 2018) concentrated more on the regulation of gut microbiota metabolites, which is manifested in reducing inflammation by enhancing the role of certain SCFAs in the intestine or increasing their production. These included propionic acid and butyric acid, which inhibited the production of inflammatory cytokines like TNF-, IL-2, and IL-6, thereby playing a role in anti-inflammatory. In the bargain, SCFAs could promote the secretion of glucagon-like peptide-1 (GLP-1) and peptide YY (PYY), hormones that helped lower blood glucose levels and improved glycolipid metabolism. Strengthening the intestinal barrier, restricting the entry of hazardous substances, controlling intestinal motility, and providing the primary energy source for colon cells are all made possible by SCFAs. At the same time, clinical trials have also achieved good results. Clinical observations indicated the anti-inflammatory and intestinal barrier-protecting properties of SCFAs, Huangkui capsule had a role in improving the gut flora’s subpopulations and dispersion by regulating the amount of gram-positive and gram-negative bacteria in the colon, Qiditang nephroid formula (QDTS) had a great effect on bile acid metabolism, and affects bile acid metabolism by changing the gut microbiota (Wei et al., 2021). Bile acids in QDTS constituted important byproduct of the metabolism of gut microbes and were essential for controlling the host’s inflammatory and metabolic reactions. Certain bile acids, such taurocholic acid (TCA), tauro-Mulisiccic acid (T-MCA), deoxycholic acid (DCA), and murichticolic acid (-MCA), was reduced in serum levels by QDTS. Bile acids were associated with the activation of FXR, which was highly expressed in the kidney. While, db/db animals treated with QDTS did not show a substantial change in FXR expression in their kidneys, QDTS may have an indirect effect on FXR activity by altering the amount of bile acids, which would protect the kidneys. San-Huang-Yi-Shen (SHYS) is a TCM formula. The study discovered that SHYS influenced the β diversity of gut microbiota in DN model rats, as well as the relative abundance and metabolite levels of particular bacterial genera. It also affected a range of metabolic pathways, including glycerophospholipid metabolic processes, tryptophan metabolic rate, alanine, aspartic acid and glutamic acid. digestion, the arginine metabolism, tricarboxylic acid (TCA) cycle, tyrosine metabolic processes, Arginine and proline metabolic rate, phenylalanine, tyrosine, and tryptophan biosynthesis, phenylalanine respiration, and D-glutamine and D-glutamate the metabolism pathways, thus ameliorate the disease. of glyconephrium (Su et al., 2021).

**TABLE 1:** Traditional Chinese medicine prescriptions improve DN by regulating the gut microbiota in experiments.

Compound prescription	Experimental model	Treatment	UP microbiota	Down microbiota	References
Zhuang medicine compound Xiancao Granule	Forty-six male Sprague Dawley (SD) rats (weight 200 ± 50 g, 5-week-old) six rats	6.3 g/kg, 3.15 g/kg, and 1.575 g/kg of CXCG, once daily for 10 weeks	Alloprevotella, Oscillibacter, Anae-roplasma,Anaerotruncus	Faecalibacterium	Mo et al. (2024)
Sanziguben polysaccharides	Six-week-old 10WT mice and 30 db/db mice	DN control group (DN), DN þ 300 mg/kg MET group (MET) and DN þ 500 mg/kg SZP group (SZP)	Phylum Verrucomicrobia Akkermansia	Proteobacteria *Klebsiella* Escherichia-Shigella Marvinbryantia	Wang et al. (2023)
Jiangtang Decoction	15 male KK-Ay mice, aged between eight to 9 weeks, and five C57BL/6J mice	4 g/kg weight of Chinese medicine JTD decoction	The genera Rikenella, Lachnoclostridium, and unclassified_c_Bacilli exhibited	norank_f_Lachnospiraceae	Hong et al. (2023)
Yuye decoction	45 6-week-old male SPF Wistar rats	5.03 g kg^-1^ of YYD extract by gavage	Bacteroidetes, *Lactobacillus*, Bifidobacterium	Firmicutes	Guo et al. (2024)
Nephritis rehabilitation tablet	7-week-old type 2 diabetic BKS-Lepr em2Cd479/Gpt mice [db/db mice, body mass (40 ± 2) g, no specific pathogens] and 8 normal mice [mage mice, body mass (18 ± 2) g, no specific pathogens	Low dose group: 1 g/kg High dose group: 2 g/kg Metformin group: 200 mg/kg Administration for 3 months		*Bacteroides* phylum	Li et al. (2018)
Suoquan Yishen prescription	db-/db-mice (normal) in a group of 8,24 db-/db-mice (DN)	db-/db-F: 0.01 mg/kg of Shrinking Spring Kidney Formula was given by gavage db-/db-IRB: 10 mg/kg Irbesartan by gavage for 8 weeks (approximately 0.5 mg/kg) db-/db-IRB: Gavage of 10 mg/kg Irbesartan for 8 weeks (approximately 0.2 mL once daily)	Cyanobacteria Verrucous microbacteria	Thick-walled bacteria,Soft-walled bacteria, Actinomycetes phylum	Yao et al. (2020)
QiDiTangShen granules	Eight-week-old male db/db mice in a C57BL/Ks background (39 ± 3 g; n = 35) and male C57BL/6 J mice (20 ± 3 g; n = 20)	QDTS: administered orally daily at a dose of 3.37 g/kg Valsartan: administered orally daily at a dose of 10.29 g/kg Treatment Cycle: All mice were treated for 12 weeks	Alloprevotella	*Lactobacillus*, *Bacteroides* Lachnospiraceae_NK4A136_group	Wei et al. (2021)
Bekhogainsam Decoction (BHID)	5-week-old male specific pathogen free (SPF) C57BL/6 mice	Low-dose BHID-treated group (100 mg/kg), high-dose BHID-treated group (500 mg/kg), and metformin-treated group (250 mg/kg). For 4 consecutive weeks	*Peptococcus*, Roseburia, Coriobacteriaceae_UCG-002, Kerstersia, Acetatifactor		Meng et al. (2020a)
Jowiseungki decoction (JSD)	5-week-old male C57BL/6 mice	JSD (100 and 500 mg/kg) treatment groups, and metformin (250 mg/kg) treatment group. Mice in the JSD treatment group were given JSD orally daily for 4 weeks	Coriobacteriaceae *Peptococcus*	Acetatifactor、Butyricicoccus、Kerstersia	Meng et al. (2020b)
Qing-Re-Xiao-Zheng formula (QRXZF)	mice	QRXZF (15.6 g/kg/day), treatment lasted 8 weeks	Rikenellaceae, Akkermansia	Desulfovibrionaceae、Desulfovibrio、Peptostreptococcaceae、Corynebacterium_1	Gao et al. (2021)
San-Huang-Yi-Shen (SHYS)	Sprague-Dawley (SD) Rats 60 male rats 6–8 weeks old, weighing approximately 200 ± 20 g	Irbesartan group SHYS low dose group SHYS medium dose group SHYS high dose group 10 rats in each group. SHYS capsules were given by gavage for 4 weeks in all groups except the control and model groups which were given distilled water	*Lactobacillus*, Ruminococcaceae UCG-005 Allobaculum Anaerovibrio, *Bacteroides* Christensenellaceae_R-7_group	Candidatus_Saccharimonas *Treponema* Desulfovibrio	Su et al. (2021)

**TABLE 2:** Traditional Chinese medicine treatments improve DN by regulating the gut microbiota in clinical.

Compound prescription	Experimental model	Treatment	UP microbiota	Down microbiota	References
Jin Gui Ren Qi Pill	45 6-week-old male SPF Wistar rats and thirty patients with diabetic nephropathy were treated at Heping Hospital, which is associated with Changzhi Medical College, between March 2021 and December 2022	the Bifidobacterium bifidum tablet group (10 patients), the Jin Gui Ren Qi Pill group (10 patients), and the control group (10 patients)	Prevotella_7		Zhang et al. (2024)
Addition and subtraction of Shenqi Dihuang decoction	A total of 160 patients in the Department of Nephrology, affiliated Hospital of traditional Chinese Medicine, Southwest Medical University from January 2018 to December 2019 were selected	control group: oral losartan potassium tablets Observation group: on the basis of treatment in the control group, Shenqi Dihuang decoction was given oral treatment. The decoction was decocted in water twice, and the solution was taken for about 400 mL. The medicine was taken twice in the morning and evening, one dose a day, and the course of treatment was 3 months	*Bacteroides*, Bifidobacterium *Lactobacillus*	Enteric bacilli Enterococci Yeast bacteria	Du X. M. et al. (2021)
Huangkui capsule	Patients with diabetic nephropathy admitted to the First People’s Hospital of Changzhou City from May 2017 to May 2019, 96 cases in total	Control group: calcium channel blocker (CCB) drugs, statins, oral hypoglycemic drugs or insulin control, and protein intake.The protein intake should be controlled at 1 g/d Experimental group: the experimental group was treated with Huangkui capsule on the basis of the control group All patients were evaluated for efficacy after 2 months of treatment	*Clostridium* tenuiformis *Lactobacillus*	*Bacteroides*, Bifidobacterium	[71]
sodium ferulate Ferula assa-foetida	Collection of patients with DN admitted to the Department of Gastroenterology DN patients admitted to the Department of Gastroenterology from April 2016 to April 2017 40 cases as study subjects	Sodium ferulate was administered as 0.2 g + 0.9% sodium chloride 250 mL intravenously once/day. Evaluation was performed after 1 month of treatment	Gram-positive bacilli (G + b) and Gram-negative bacilli (G-b)	Cocci/bacilli ratio (c/b)	Zheng et al. (2011)

### 5.2 Traditional Chinese medicine ingredients

The active ingredients are the target site for the effectiveness of TCM. Various TCM ingredients were identified by morden technology and were used for conducting experiments on the treatment of DN (Table 3; Table 2). The composition of corn silk polysaccharides (CSPS) has the potential to enhance the gut microbiota’s structure (Dong et al., 2023). Patients suffering from DN frequently have an imbalance in their gut microbiota. CSPs can regulate the dominant strains in the gut, such as Firmicutes, Bacteroidetes, Lachnospiraceae-NK4A136 and Dubosiella. Meanwhile, CSPs can affect the metabolism of key endogenous metabolites that interact with the gut microbiota, including glycerophosphate, fatty acids, bile acids, tyrosine, tryptophan, and phenylalanine. Variations in these metabolites are directly linked to the onset of DN and are linked to changes in the gut flora. Among these, the intestinal microbiota has a major impact on bile acid metabolism. CSPs can control bile acid metabolism and minimize bile acid buildup in the body, which lowers the inflammatory response and kidney cell damage. Peony peel polysaccharide (MC-Pa) improves the pathological condition of DN by influencing the gut microbiota and its byproducts through several means. It can improve the structure of the intestinal microbiota and enhance the intestinal barrier function, among which it is worth noting that MC-Pa can promote the production of SCFAs and reduce the production of branched-chain fatty acids (BCFAs), thereby exerting a positive therapeutic effect on DN (Zhang M. et al., 2022). In addition to the regulation of the structure of the gut microbiota and its metabolites, cinnamaldehyde, rehmannia leaf glycosides, Yam polysaccharides and total phenolic acids were all effective in improving renal function and regulating the gut microbiota (Zhang et al., 2021; Xu et al., 2021; Dai et al., 2017; Wang et al., 2018). Magnesium lithospermate B (MLB), a drug used to treat angina, has been found to improve kidney damage in a mouse model of DN via alterations to the bile acid biosynthesis and gut microbiota, in addition to which MLB may indirectly improve kidney function by affecting microbiota associated with blood pressure regulation (Zhao et al., 2019). But, the results on experimental animals can not completely represent the clinical effects. Surprisingly, a clinical study shown that sodium ferulate have great potential in the management of DN patients by alterations to the gut microbiome (Zheng et al., 2011). On the basis of animal experiments, it is believed that more TCM active ingredients will also be able to be applied in clinical practice.

**TABLE 3:** Traditional Chinese medicine ingredients improve DN by regulating the gut microbiota in experiments.

Active ingredients	Source	Experimental model	Treatment	UP microbiota	Down microbiota	References
Corn silk polysaccharides	Corn silk	85 male SD rats weighing 200 ± 20 g	During an 8-week continuous intervention, NC and DN received 20 mL/kg of distilled water by gavage, 0.25 g/kg of metformin by gavage, and dose concentrations of polysaccharides (ranging from low to high) by gavage: 100 mg/kg, 200 mg/kg, and 400 mg/kg	Lachnospiraceae-NK4A136 group Dubosiella	Firmicutes Bacteroidota Fusobacteriota	[72]
Moutan Cortex polysaccharide	Moutan Cortex	3-week-old male SD rats	MC-Pa low dose group (MC-Pa-L, 80 mg/kg BW, n = 15) MC-Pa high dose group (MC-Pa-H, 160 mg/kg BW, n = 15) Aminoguanidine (Aminoguanidine, AG, as positive control, 100 mg/kg BW, n = 15) Animals in each group were administered daily by gavage for 12 weeks	*Lactobacillus* Akkermansia Muribaculaceae_unclassified	Clostridiales_unclassified Ruminococcus_1 Alistipes	Dong et al. (2023)
yam polysaccharide	Dioscorea opposita	Sixty clean-grade SD rats, half male and half female, 7–9 weeks of age, weighing 180–220 g were used	Normal group (8 rats fed on basal chow, anesthetized intraperitoneally only, injected with saline after exposing the left kidney) Model group (given equal amount of saline by gavage) Positive drug group (given 10 mg/kg lodinexin by gavage) Yam polysaccharide administration group was given 50 mg/kg, 100 mg/kg, 200 mg/kg respectively Treatment was continued for 30 days, once daily	Firmicutes Lachnospiraceae Veillonella *Bacillus* Paenibacillus Acidobacterium	Bacteroidetes Proteobacteria *Bacteroides* *Escherichia* *Shigella* *Salmonella*	Zhang M. et al. (2022)
Total Phenolic Acid from the Stems and Leaves of Salvia miltiorrhiza Bge	Salvia miltiorrhiza Bge	7-week-old male spontaneous type 2 diabetic and obese db/db mice	Positive drug metformin group (MH) Salvia miltiorrhiza stem and leaf phenolic acid low dose group (JL) high dose group (JH) Danshen root phenolic acid low dose group (GL) high dose group (GH)	g_Lachnoclostridium g_Eubacterium_nodatum_group g_Lachnospiraceae_UCG-001 g_Rikenellaceae_RC9_gut_group g_Ruminococcaceae_UCG-013 g_Eubacterium_coprostanoligenes_group - g_Ruminiclostridium_5 g_*Lactobacillus*	g_unclassified_f__Coriobacteriaceae g_unclassified_p__Firmicutes g_Bacteroidales_S24-7_group	Zhang et al. (2021)
Cinnamaldehyde	Cinnamomum verum	Healthy male Sprague Dawley rats were 8 weeks old and weighed 200–250 g	Gavage with cinnamaldehyde every day from day 4 (2.1 mL of cinnamaldehyde in 540 mL of distilled water, 3 mL/day only).The observation time points were 4 weeks, 8 weeks and 12 weeks after modeling	*Lactobacillus* Alloprevotella	Sutterella *Bacteroides*	Dai et al. (2017)
total glycoside extracted from leaves of Rehmannia (TLR)	Rehmannia glutinosa Libosch	SD rat, male, body mass about (220 ± 20) g	Wasabi capsule group (HK): the administered dose was 0.75 g-kg-1-day-1 Irbesartan group (YX): the administered dose was 27 mg-kg-1-day-1 Total Digitonin Leaf Extract Low Dose Group (DHYL), High Dose Group (DHYH): the administered dose was 4.3 g-kg-1-day-1, 7.2 g-kg-1-day-1, respectively Total Glycosides of Digitonin Leaf Capsules Low Dose Group (JNL), High Dose Group (JNH): the administered dose was 216 mg-kg-1-day-1, 360 mg-kg-1-day-1, respectively	Bifidobacterium,Transmissibacterium, Streptococcaceae, Desulfovibrio, SMB53	*Lactobacillus*, *Clostridium*, Richenobacteriaceae, Rumatobacteriaceae	Xu et al. (2021)
Magnesium lithospermate B (MLB)	Salvia miltiorrhiza	DBA/2J mouse	MLB: 50 mg/kg Treatment lasted 8 weeks	Odoribacter	*Escherichia*, *Shigella*, *Proteus*, NK3B31, Marvinbryantia, Ruminiclostridium 6	Wang et al. (2018)

### 5.3 Single herb Chinese medicine

When treating DN, single herb Chinese medicine is also effective in controlling the gut flora and its metabolites (Table 4). For example, Danshen from the roots of Salvia miltiorrhiza plants can alleviate the glucose kidney lesions by increasing the relative microbial abundance. In addition, the stems and leaves of Salvia miltiorrhiza are usually discarded as waste, which were proved to have certain pharmacological activities to regulate gut microbiota, providing a basis for the comprehensive utilization of Salvia miltiorrhizae plants (Xu et al., 2021; Cai et al., 2021). It can be seen that Salvia miltiorrhiza has an important role in the treatment of DN, by regulating the interaction between the gut microbiota and metabolites, such as phosphatidylcholine (PC), phosphatidylethanolamine (PE), lysophosphatidylcholine (LysoPC), sphingomyelin (SM), and other metabolites. Its complex mode of action involves not just interacting with metabolites or gut microbiota individually, but also regulating the balance of the gut microbiota as a whole and the interaction with individual microorganisms (Gill et al., 2006). Dendrobium can not only improve the abundance of gut microbiota, but also increase the relative abundance of beneficial bacteria such as Prevotella/Akkermansia, and reduce the relative abundance of harmful bacteria such as S24-7/Rikenella/*Escherichia coli*, which may be associated with inflammation and insulin resistance. It also impacts the synthesis of fatty acids, such as SCFAs, which can modify lipid metabolism and lower dangerous levels of cholesterol and triglycerides while improving the barrier function of colonic epithelial cells and reducing inflammation (Li et al., 2018). However, there is no clinical research on a single herb Chinese medicine. These studies are still in the animal experimental stage, and clinical trials and applications are worth looking forward.

**TABLE 4:** Single herb Chinese medicine improves DN by regulating the gut microbiota in experiments.

Single substance	Experimental model	Treatment	UP microbiota	Down microbiota	References
Salvia miltiorrhiza	SPF grade male rats with body weight in the range of (220 ± 20) g	Irbesartan Wong Kwai Capsules Different dosage administration groups of Salvia divinorum root and rhizome and Salvia divinorum stem and leaves: including aqueous and alcoholic extracts in low, medium and high doses	Turicibacter, Bifidobacterium, Peptostreptococcaceae, Desulfovibrio	*Lactobacillus*, *Clostridium*, Rikenella, Rumen fungi	Zhao et al. (2019)
Alpinia oxyphylla Miq	mice	The DB-/DB-S group and the db-/db-S group were given only saline in an amount equal to the drug.The db-/db-YZR and db-/db-IRB groups were administered 1.5 mg/g of Pueraria Mirifica and 10 mg/kg of Irbesartan by gavage for 8 weeks, respectively	Cyanobacteria, Deferribacteres, Verrucomicrobia, Intestinimonas, Mucispirillum, Parabacteroides, Alistipes Akkermansia, Ruminococcaceae_UCG-007,Prevotellaceae_Ga6A1_group, Anaerotruncus. Ruminococcaceae_UCG-009,*Bacteroides*	Corynebacteriaceae, Alcaligenaceae, Aerococcaceae, Carnobacteriaceae, Trichococcus, Oligella Aerococcus, Vagococcus Parasutterella, Erysipelatoclostridium, Corynebacterium_1, Facklamia	Cai et al. (2021)
Dendrobium	Eight mice of C57BL/6 (male, 6–8 weeks old) and forty mice of BKS.Cg-Dock7m+/+Leprdb/Nju (db/db mice, male, 6–8 weeks old)	Three dose groups of DJP were used for treatment, (25 mg/kg), (50 mg/kg) and (100 mg/kg). It lasted for 8 weeks	Prevotellaceae, Akkermansia muciniphila	*Escherichia* S24-7, Rikenella	Gill et al. (2006)

## 6 Summary

This review summarized the gut microbiota and its metabolites on affecting DN, and introduced the effects and therapeutic promise of various TCM prescription, Chinese herbal medicine and active TCM ingredients on gut microbiota to further ameliorate DN through microbiota modulation. With the improvement on the theory of the intestinal-renal axis, more and more researchers are paying attention to the metabolites of the gut microbiota, and for DN. TCM as a therapeutic tool have the potential on interfering the intestinal tract and DN, with the characteristics of multiple pathways, multiple targets, and overall regulation. And the intervention of TCM on gut microbiota and its metabolites has a positive impact on DN prognosis, which also suggests that gut microbiota and its metabolites are important therapeutic targets for DN. It is crucial to acknowledge the predominant reliance on preclinical models in current research. The vast majority of mechanistic insights and efficacy data summarized derive from rodent studies, providing essential but preliminary understanding of microbial shifts and TCM’s multi-target actions. In stark contrast, robust clinical validation remains limited, with only a small number of human trials exploring specific TCM interventions like Huangkui capsule or modified Shenqi Dihuang decoction. These clinical studies, while indicative of potential, are often constrained by small sample sizes, lack of standardized TCM preparations, and variable dosing regimens, highlighting a significant translational gap. To effectively bridge this gap and realize the therapeutic potential of TCM in DN treatment, it is necessary to implement comprehensive chemical fingerprinting to define and quantify key bioactive constituents to provide more scientific foundation for dosage conversion and optimization, and ensure the transformation and reproducibility from preclinical experimentation to clinical trials. Future preclinical work should systematically establish dose-response relationships and conduct thorough pharmacokinetic/pharmacodynamic profiling to identify effective and safe dosage ranges, facilitating rational translation to human studies rather than relying on empirical or weight-based scaling. Furthermore, meticulous attention to long-term safety monitoring, particularly regarding potential herb-drug interactions in a population often on complex polypharmacy, is paramount.

In conclusion, while TCM modulation of the gut-kidney axis presents a compelling, multi-faceted strategy against DN, its successful translation hinges critically on addressing the current disparity between promising preclinical findings and insufficient clinical validation. By mandating rigorous standardization of TCM content and dosages, establishing evidence-based dosing through advanced pharmacokinetic/pharmacodynamic modeling, and conducting high-quality clinical trials with integrated biomarker analysis, the field can generate robust, reproducible data. This disciplined approach is essential to move beyond preliminary observations and establish TCM-derived microbiota modulators as credible, effective therapeutic options for diabetic nephropathy patients. Therefore, it is hoped that more researchers will take part in this study, opening up new avenues for the treatment of DN using natural medicines from TCM.

## Abbreviations

DN, diabetic nephropathy; TCM, traditional Chinese medicine; IL, interleukin; TNF-α, tumor necrosis factor-alpha; ROS, reactive oxygen species; NF-κB, nuclear factor kappa-light-chain-enhancer of activated B cells; SCFA, short-chain fatty acids; FMT, fecal microbiota transplantation; AKK, Akkermansia; BBR, berberine; PPAR-γ, peroxisome proliferator-activated receptor gamma; SGLT2, sodium-glucose cotransporter-2; RAS, renin-angiotensin system; GPR, G protein-coupled receptor; AMPK, adenosine monophosphate-activated protein kinase.
